# Event‐related Theta Phase Coherence differ between stable and progressive MCI: aTwo‐Year Follow‐up study

**DOI:** 10.1002/alz.090683

**Published:** 2025-01-09

**Authors:** Yağmur Özbek, Görsev Yener

**Affiliations:** ^1^ Department of Neurosciences, Health Science Institute, Dokuz Eylül University, Izmir Turkey; ^2^ Izmir Biomedicine and Genome Center, Izmir Turkey; ^3^ Izmir University of Economics, Faculty of Medicine, Balçova, Izmir Turkey

## Abstract

**Background:**

Mild cognitive impairment (MCI) is viewed as a transitional stage between normal aging and Alzheimer's disease (AD), serving as a pathological precursor to AD. Longitudinal studies indicate an annual conversion rate from MCI to AD of 10‐15%. Our previous studies have shown decreased coherence values in visual event‐related slow frequency bands including theta frequency. This study aimed to compare event‐related theta inter‐trial phase coherence (ITC) in two groups of MCI, stable or progressive MCI, and cognitively unimpaired (CU) seniors, during a two‐year follow‐up.

**Method:**

Out of 33 individuals with MCI, 14 of the MCI patients progressed into AD dementia (ADD) at a two‐year follow‐up (progressive MCI: pMCI), whereas 19 MCI patients remained stable (stable MCI: sMCI). A total of 34 cognitively unimpaired (CU) seniors were included in this study. Classical visual oddball paradigm was used to elicit event‐related (ER) oscillations. Continuous Wavelet Transformation was used for intertrial phase locking (Intertrial Coherence) analyses.

**Result:**

Mixed‐design ANOVA analysis showed TIME x LOCATION x GROUP interaction effect in theta ITC. Posthoc analyses indicated baseline temporal theta ITC values of CU were higher than sMCI (p = 0.028) and pMCI (p = 0.014). In addition, follow‐up measurements of central theta ITC of CU were higher than pMCI (p = 0.033), and parietal theta ITC of CU was higher than pMCI (p = 0.008) and sMCI (p = 0.002). It was found that the baseline temporal ITC values of CU (p = 0.018) and the baseline parietal theta ITC values of sMCI (p = 0.033) were higher compared to follow‐up measurements.

**Conclusion:**

Among the classical frequency bands, the stimulus‐locked ER theta phase reflects best about temporal processes of cortical functions for the MCI patients who convert to dementia. Furthermore, the increased ER theta coherence at the stable MCI level returns to the normal values at the later stages of MCI (pMCI). Our findings are in line with the hyperconnectivity seen at the stable MCI stage, even before becoming progressive MCI. Theta ITC may provide electrophysiological cues for displaying clinical stages during MCI period.

